# Climate of Aspley Guise

**Published:** 1859-01

**Authors:** 


Topography of Aspley Guise.

A Notice of Dr. James Williams's work on the *Topography and Climate of Aspley Guise in reference to their Influence upon Health and Disease*, as it appeared in its first edition, has already appeared in the pages of the SANITARY REVIEW. In this second and enlarged edition, Dr. Williams gives a more detailed account of the locality concerning which he writes, including some most interesting details, geographical, geological, and botanical. The facts relating to climate are also described with great care; and comparisons are drawn between the climate of this spot and the climates of Nice, Pau, Madeira, the Isle of Wight, Hastings, and the Channel Islands. In a word, we are of opinion that Dr. Williams's descriptions of an English resting place for the English man or woman whose physical impairments demand rest, pure air, and the renovating agency of nature seen in her most enticing vestments, cannot be made too well or widely known. In the last part of his work, Dr. Williams gives an outline of the hygienic treatment of pulmonary consumption, in which he supports, at considerable length, the views which have been often advocated in this Review.

